# Comprehensive analysis of aberrantly expressed circRNAs, mRNAs and lncRNAs in patients with nasopharyngeal carcinoma

**DOI:** 10.1002/jcla.24836

**Published:** 2023-01-04

**Authors:** Feifan Guo, Dayang Chen, Zengyan Zong, Wei Wu, Chan Mo, Zhou Zheng, Jian Li, Xiuming Zhang, Dan Xiong

**Affiliations:** ^1^ School of Medicine Anhui University of Science and Technology Huainan China; ^2^ Medical Laboratory of the Third Affiliated Hospital of Shenzhen University Shenzhen China; ^3^ Department of Otolaryngology, The First Affiliated Hospital Sun Yat‐sen University Guangzhou China; ^4^ Guangzhou Key Laboratory of Otorhinolaryngology Guangzhou China

**Keywords:** expression profiles, nasopharyngeal carcinoma, prediction model, regulatory mechanisms

## Abstract

**Background:**

The location of nasopharyngeal cancer is hidden, so it is difficult to diagnose at an early stage. In this study, we aimed to investigate the expression profiles of circRNAs, mRNAs and IncRNAs and to provide some basis for further studies.

**Methods:**

Expression profiles of circRNAs, mRNAs, and lncRNAs were analyzed using microarray techniques. The differentially expressed ncRNA was calculated by bioinformatics.

**Results:**

A total of 3048 circRNAs, 2179 lncRNAs, and 2015 mRNAs were detected to be significantly differentially expressed in NPC. The most upregulated circRNAs, lncRNAs, and mRNAs were hsa‐circ‐0067562, NONHSAT232922.1, and HOXB13, respectively. And, the most downregulated circRNAs, lncRNAs, and mRNAs were hsa_circ_0078837, lnc‐TTC8‐4:3, and LTF, respectively. The number of upregulated DE lncRNAs was more than twice than those downregulated. Our data showed that 80.44% of pairs of lncRNAs and cis‐mRNAs demonstrated positive correlations. For lncRNAs and trans‐mRNAs pairs, 53.7% of pairs showed positive correlation. LncRNA‐mediated cis regulation is a prevalent regulatory mode in the development of nasopharyngeal carcinoma. CR1, LRMP and SORBS2 are predicted to be mediated not only by cis‐acting lncRNA modes of action, but also by trans‐acting lncRNA mechanisms. Additionally, we constructed a diagnostic prediction model with a high sensitivity and specificity.

**Conclusion:**

Our study characterized the landscape of circRNAs, mRNAs and lncRNAs in NPC tissue and provided novel insights into the molecular mechanisms of NPC.

## Graphical abstract

In this study, we aimed to investigate expression profiles of circRNAs, mRNAs, and lncRNAs and to provide some basis for further study. The expression profiles of circRNAs, mRNAs, and lncRNAs were analyzed by microarray technology. The differentially expressed ncRNA was calculated by bioinformatics. A total of 3048 circRNAs, 2179 lncRNAs, and 2015 mRNAs were detected to be significant differentially expressed in NPC. The number of upregulated DE lncRNAs was more than twice than those downregulated. Our data showed that 80.44% lncRNAs and cis‐mRNAs pairs demonstrated positive correlation. For lncRNAs and trans‐mRNAs pairs, 53.7% pairs showed positive correlation. LncRNA‐mediated cis regulation is a prevalent regulatory mode in the development of nasopharyngeal carcinoma. CR1, LRMP, and SORBS2 are predicted to be mediated not only by cis‐acting lncRNA modes of action, but also by trans‐acting lncRNA mechanisms. Additionally, we constructed a diagnostic prediction model with a high sensitivity and specificity. Our study characterized the landscape of circRNAs, mRNAs, and lncRNAs in NPC tissue and provided novel insights into the molecular mechanisms of NPC.

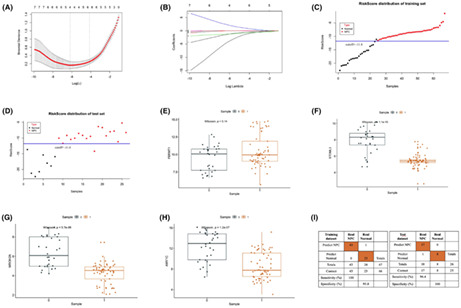

## 1 INTRODUCTION

Nasopharyngeal carcinoma (NPC) is characterized by extremely unbalanced geographical global distribution and more than 70% of new cases occur in east and southeast Asia.^1^ Multiple factors, including Epstein–Barr virus (EBV) infection, environmental factors, genetic susceptibility, and ethnic background, are contributors to NPC progression.^2^ Because NPC primary site is located in a cryptic area at early stage, the majority of clinical patients have reached a more advanced stage, with a 5‐year survival rate of 50% to 60%.^3^ Therefore, it is most required to identify effective molecular biomarkers for the early diagnosis, prognosis prediction, and therapeutic treatment.

Long noncoding RNAs (lncRNAs) are nonprotein coding transcripts with the length over 200 nucleotides. Several supporting evidences suggested that lncRNAs, by a variety of mechanisms, play modulator roles in many biological processes. Some studies have identified that the dysregulated lncRNAs have a significant role in NPC.^4^, ^5^ LncRNAs can regulate the expression of their corresponding target genes by *cis* and *trans*‐acting modes^6^ or interacting with mRNA, DNA, protein, and miRNA. For instance, several lncRNAs, including *PCAT7*,^7^ *LncRNA‐LINC0046*,^8^ *LOC100129148*,^9^ and *CCAT1*,^10^ regulate NPC tumorigenesis by targeting microRNA. Ma et al.^11^ reported that upregulation of lncRNA HOTAIR contributes to the tumorigenesis of NPC via upregulating fatty acid synthase.

Circular RNAs (circRNAs) were previously considered to be covalently linked noncoding RNAs obtained by back‐splicing exons from a single pre‐mRNA. Recent study demonstrates that some circRNAs have translation functions. There is increasing evidence that circNRAs can be divided into noncoding circrnas and coding circrnas.^12^, ^13^ Because circRNAs is a kind of singled‐stranded RNA with neither 5′‐3′ polarities nor polyadenylated tails, it is refrained from exo‐nucleolytic degradation and more stable.^14^, ^15^, ^16^ Due to those characteristics, circRNAs are regarded as a promising biomarkers. Several supporting evidences have shown that circRNAs with miRNA response elements (MREs) competitively bind to miRNAs to regulate target genes expression.^17^, ^18^ Recently, several circRNAs were reported to function as miRNA sponge in the progression of NPC.^19^, ^20^, ^21^, ^22^

To the best of our knowledge, system identification of circRNAs, mRNAs and lncRNAs and exploring their complex regulatory interaction in NPC remain unclear. In this research, four NPC tissue samples and four chronic nasopharyngitis tissue samples were assessed by microarray analysis. Then, we investigated the differential expression patterns of circRNAs, mRNAs, and lncRNAs and associated pathways as well as gene ontology items. Subsequently, circRNA‐miRNA, circRNA‐mRNA, and lncRNA‐mRNA interaction networks were constructed. In addition, the *cis* and *trans* target genes of lncRNA were predicted. These findings provided a novel basis that circRNAs, mRNAs, and lncRNAs could serve as potential biomarkers, and the information generated from these predictions could be utilized in further studies.

## 2 MATERIAL AND METHODS

### 2.1 Tissue samples

Fresh tissue samples of NPC (*n* = 4) and the chronic inflammation of the nasopharyngeal mucosa tissues (*n* = 4) were obtained from the the First Affiliated Hospital of Sun Yat‐sen University hospital. This study was approved by the First Affiliated Hospital of Sun Yat‐sen University hospital. Experimental tissue specimens were diagnosed based on rigorous pathologic examination. They had no other history of tumors and had not undergone radiotherapy and chemotherapy. Basic information is presented in Table 1. The correlation analysis among samples is shown in Figure S1.

**TABLE 1:** Detailed information about the tissue samples included in the microarray analysis.

	Age	Gender	Type	Stage	EBV‐DNA	EBV‐IgA	EBV‐IgM	EBV‐IgG
T1	67	F	Tumor	T1N0M0 (I Stage)	<500	P	N	P
T2	51	M	Tumor	T3N1MO (III Stage)	<500	N	N	P
T3	28	F	Tumor	T3N2MO (III Stage)	9.2 × 10^2^	N	N	P
T4	30	F	Tumor	T4N1M0 (IV Stage)	<500	N	N	P
N1	41	M	Control	/	<500	N	N	P
N2	38	F	Control	/	<500	N	N	P
N3	50	F	Control	/	<500	N	N	P
N4	23	F	Control	/	<500	P	N	P

Abbreviations: Control, Chronic inflammation of the nasopharyngeal mucosa; F, Female; M, Male; N, Negative; P, Positive; Tumor, Nonkeratinizing undifferentiated NPC.

### 2.2 RNA isolation and quality control

Total RNA was extracted from the frozen tissue specimens using TRIzol reagent (Invitrogen, Grand Island, NY, USA) according to the manufacturer's instructions, and purified by using a RNeasy Mini Kit (Qiagen, GmBH, Germany). Total RNA was checked for a RNA integrity number (RIN) to inspect RNA integration by an Agilent Bioanalyzer 2100 (Agilent technologies, Santa Clara, CA, US). The quality and amount of RNA were assessed using a NanoDrop ND‐2000 spectrophotometer (Thermo Scientific, Rockford, IL, USA).

### 2.3 Microarray analysis

Sino Human ceRNA array V3.0 (Shanghai Sinomics Corporation, China), which contains 53,635 circRNAs, 91,614 lncRNAs, and 25,353 mRNAs, was performed to detect the genome‐wide profiling. RNA samples from each group were used to generate biotinylated cRNA targets. The labeled cRNA targets were then hybridized with the slides. Agilent Microarray Scanner (Agilent Technologies, Santa Clara, CA, USA) was performed to scan the slides. Feature extraction software 10.7 (Agilent Technologies, Santa Clara, CA, USA) was carried out to extract the raw data. The microarray experiments were performed by following the protocol of Agilent Technologies Inc.

### 2.4 Differential expression analysis

The raw data were normalized by the quantile algorithm, R package “limma.” |log fold change (FC)| > 1.0 and *p*‐value <0.05 were conducted to indicate a significant circRNAs, lncRNA, and mRNA difference. The differentially expressed mRNAs were selected to perform Gene Ontology (GO) database and the functional pathways (KEGG) analysis.

### 2.5 Co‐expression networks and competitive endogenous RNA (ceRNA) network analysis

We predicted the MREs of differentially expressed circRNAs and constructed a circRNA‐miRNA and circRNA‐mRNA networks with the Arraystar miRNA target prediction software (Arraystar, MD, USA). Interactions of circRNAs with putative complementary miRNAs were presented with Cytoscape 3.7.2. Pearson correlation coefficients (PCCs) were used to construct lncRNA‐mRNA networks.

### 2.6 qRT‐PCR validation

Total RNA was extracted from NP69, HNE1, HONE1, and 6‐10B cultured cells by RNAiso Plus (Takara, China). All cell lines were a gift from Musheng Zeng (Cancer Center of Sun Yat‐sen University). NP69 cells grown in keratinocyte/serum‐free (KSF) medium (Invitrogen). HNE1, HONE1, and 6‐10B cells were grown in RPMI‐1640 medium (GIBCO) supplemented with 5% (vol/vol) fetal bovine serum (FBS, GIBCO). All cells were cultured in an incubator at 37°C with humidified 5% (vol/vol)CO_2_. The cDNA was used as the template with SYBR Green PCR Master Mix, and in triplicate subjected to PCR. An average Ct value from the triplicate assays was used for further calculations, and all the samples were normalized to the signal generated by GAPDH.

The primer sequences were as follows:

hsa_circ_0002354:F,5′‐ATGAATGGCTGCGAGAAGGA‐3′
hsa_circ_0002354:R,5′‐TAGTGGAATAGGCCCTGGGAA‐3′
hsa_circ_0055854:F,5′‐GAAAGGTGATGCAGAGCCAG‐3′
hsa_circ_0055854:R,5′‐GCAGAAAGTGGTGGAAGCC‐3′
GAPDH: F,5′‐AGAAGGCTGGGGCTCATTTG‐3′;
GAPDH: R,5′‐GCAGGAGGCATTGCTGATGAT‐3′.

The relative expression levels were determined by the 2^−△△Ct^ method.

### 2.7 Prediction of the potential target genes of DE lncRNAs

LncRNAs could negatively or positively regulate mRNA/protein‐coding gene expression by *cis‐* or *trans‐*acting modes. All genes within 10 kb of the DE lncRNAs were picked out as *cis‐*target genes. Bioinformatics analysis was used to search for the potential *trans*‐acting genes. We used BLASTN to search potential target mRNA sequences complementary to the lncRNA sequences. Then, lncRNA‐mRNA duplex energy was calculated by RNAplex software.

### 2.8 Building a diagnostic model

Three datasets (GSE64634, GSE53819, and GSE12452) retrieved from the Gene Expression Omnibus database (http://www.ncbi.nlm.nih.gov/geo/) included 61 NPC samples and 32 normal samples. All samples were randomly split into training and validation cohorts with a 7:3 ratio, corresponding to 67 and 26 total samples, respectively. To identify potential diagnosis indicators of NPC, the top nine dysregulated mRNAs in our microarray data set were analyzed. With the implementation of the R package of “glmnet” methods, we applied least absolute shrinkage and selection operator (LASSO) for building a diagnostic model on the training dataset. The tuning parameter was determined according to the binomial deviance estimated from 10‐fold cross‐validation, and we adopted the lowest binomial deviance, known as “lambda.min.” The difference in RiskScore distribution was examined by Wilcoxon rank sum test.

## 3 RESULTS

### 3.1 Identification of circRNAs, lncRNAs, and mRNAs differentially expressed in NPC

**FIGURE 1:**
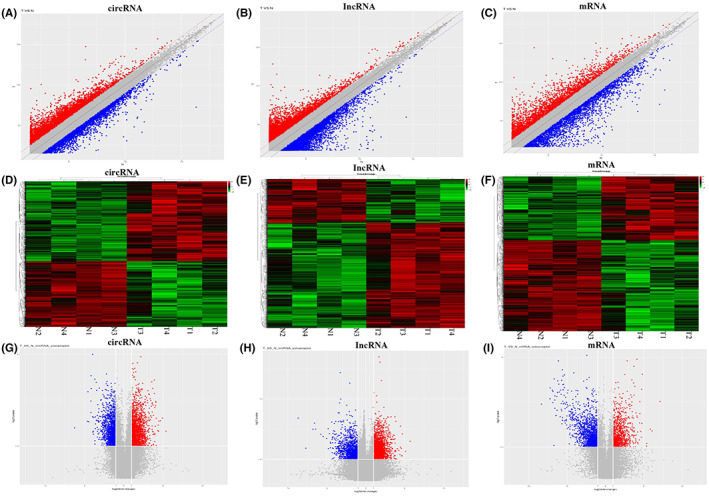
Expression levels of DE circRNAs, lncRNAs, or mRNAs in NPC tissues compared with the chronic nasopharyngitis tissues. (A–C) A volcano plot revealed differentially expressed RNAs with various p‐values and fold changes in NPC tissues compared with the chronic nasopharyngitis tissues. The values plotted on the *x*‐ and *y*‐axes are the averaged normalized signal values of the samples (log2 scale). The central gray dots indicate no difference between the two groups. The red dots above the top line and blue dots below the bottom line have >twofold change between the two groups. (D–F) Hierarchical clustering analysis illustrated the expression amounts heat map. Each row represents a single circRNAs, lncRNAs, or mRNAs, and each column represents one sample. Red indicates high relative expression, and green indicates low relative expression. (G–I) The scatter plot assesses the overall distribution of normalized signal values. The red dots indicate upregulated genes, blue dots represent downregulated genes, and gray dots represent no differentially expressed genes. N: chronic nasopharyngitis tissues; T: NPC tissues.

A total of 3048 circRNAs, 2179 lncRNAs and 2015 mRNAs were detected to be significant differentially expressed (DE) (|log fold change | > 1.0, *p* value <0.05). The significant differentially expressed circRNAs included 1674 upregulated and 1374 downregulated circRNAs. In addition, 1540 and 639 lncRNAs were upregulated and downregulated, respectively. Eight hundred thirty‐two and 1183 mRNAs were upregulated and downregulated in NPC tissues compared with the chronic nasopharyngitis tissues. The most upregulated and downregulated circRNAs, lncRNAs, and mRNAs are listed in Table 2. The volcano plots and clustering plots data suggested that the expression of circRNAs, mRNAs and lncRNAs in NPC tissues differed from that of control tissues (Figure 1).

**TABLE 2:** Significant differentially expressed RNAs in NPC tissues compared with the chronic nasopharyngitis tissues (|log fold change | > 1.0, *p* value <0.05)

Category	No. of upregulated	No. of downregulated	Total No.	The most upregulated (fold change)	The most downregulated (fold change)
circRNA	1674	1374	3048	hsa_circ_0067562	hsa_circ_0078837
lncRNA	1540	639	2179	NONHSAT232922.1	lnc‐TTC8‐4:3
mRNA	832	1183	2015	HOXB13	LTF

**FIGURE 2:**
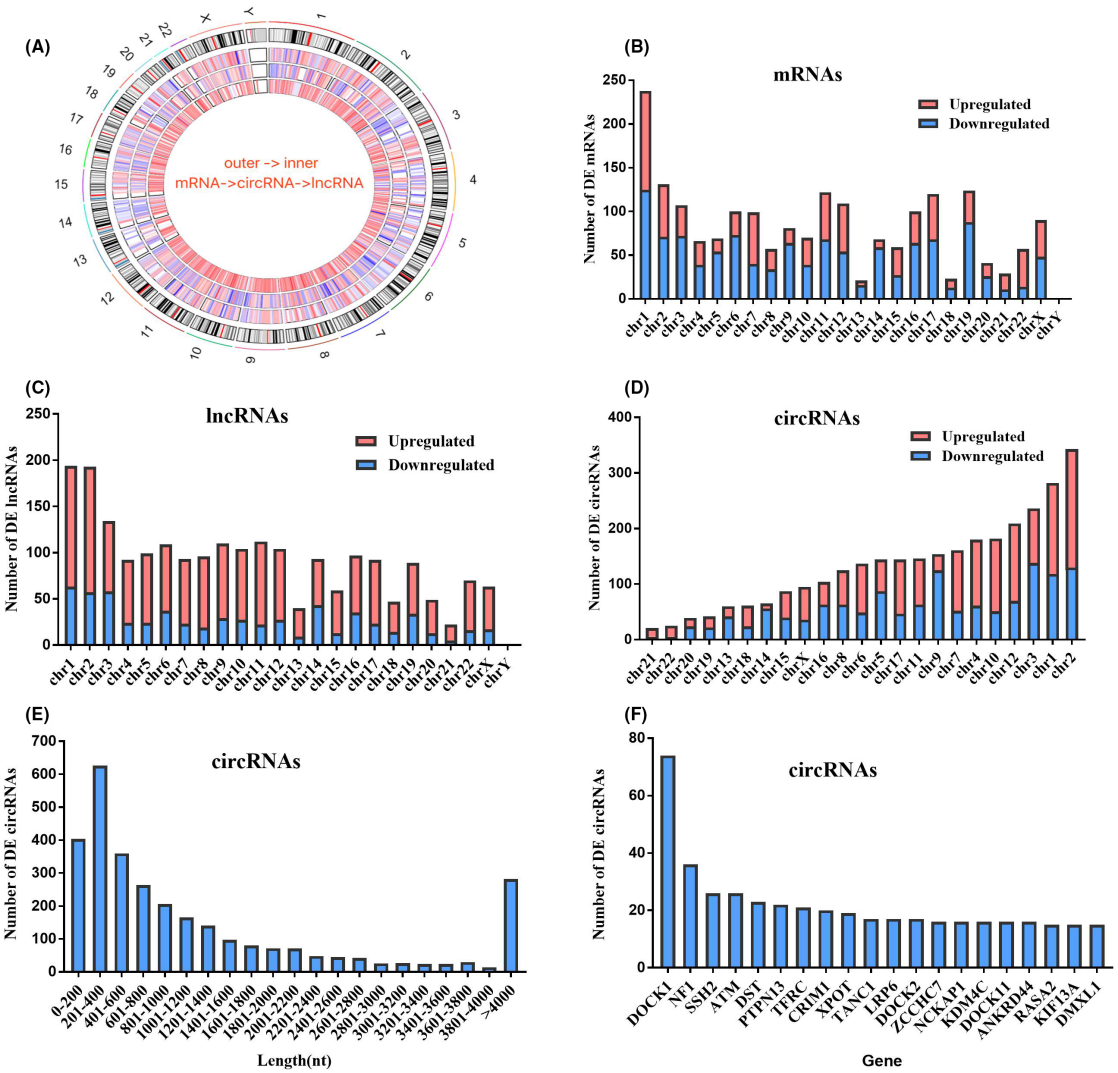
Basic characteristics of mRNAs, circRNAs, and lncRNAs. (A) Using circos plot to visualize differentially expressed RNA in all chromosomes. Chromosome numbers and bands are displayed in the outer‐most ring. Other tracks from outer to inner represent: differentially expressed mRNAs, circRNAs, and lncRNAs according to their chromosome locations. Colors from blue (lowest logFC) to white then red (highest logFC). (B–D) The distribution of the detected differentially expressed RNA in human chromosomes. (E) The spliced lengths of the differentially expressed circRNAs. (F) The genes that at least correspond to 15 DE circRNAs.

Some general characteristics of differentially expressed RNAs were summarized to understand the overall expression profile. As shown in Figure 2A, the DE RNAs were widely distributed in all chromosomes. The number of downregulated DE mRNAs was larger than that of unregulated DE mRNAs. The number of upregulated DE lncRNAs was more than twice than downregulated DE lncRNAs (Figure 2C). The number of DE circRNAs on chromosome 2 was larger than any others, suggesting that chromosome 2 might play an important role in pathogenesis. Sequence length analysis revealed that circRNA spliced length were mostly 200–400 nt. Approximately 79.23% of the circRNAs had a predicted spliced length of <2000 nt, including 45.6% of circRNAs with <600 nt, 61.02% of circRNAs with <1000 nt, and 74.21% of circRNAs with <1600 nt. Furthermore, more attention should be paid to these genes that at least correspond to 15 DE circRNAs (Figure 2F). Especially the DOCK1 gene, its circRNAs number is much higher than other genes.

### 3.2 GO and KEGG pathway analysis in these DE RNAs

Predicted GO terms and KEGG pathways with P‐values of less than 0.05 were chosen. Gene Ontology categories include biological process (BP), cell component (CC), and molecular function (MF). The top 30 GO terms of the DE RNA are displayed in Figure S2 and ranked by “Rich factor.” Gene Ontology analysis has demonstrated that the most enriched GO analyses of mRNAs are mainly related to tolerance induced self‐antigens in BP, troponin complexes in CC, and CXCR3 chemokine receptor binding in MF. With regard to GO analysis of DE circRNA host genes, the most enriched GO terms are transforming growth factor‐beta secretion in BP; the top terms in CC was kinetochore microtubule; in MF, coreceptor activity involved in *Wnt* signaling pathway was the most enriched GO terms. In terms of GO analysis of DE lncRNAs, the potential *trans*‐target and *cis*‐target genes were used to perform GO enrichment analyses. The top *trans*‐target gene GO terms include L‐methionine salvage, L‐serine transmembrane transporter activity, and Cul4A‐RING E3 ubiquitin ligase complex. The top *cis*‐target gene GO terms contain glossopharyngeal nerve development, clathrin coat of coated pit, and calcium‐release channel activity.

The top 30 KEGG pathways of the DE RNA are displayed in Figure S3. We conducted KEGG enrichment analysis for mRNA, circRNA‐hosting genes, lncRNA‐*trans*‐target and *cis*‐target genes, and the corresponding top KEGG pathways were D‐Glutamine and D‐glutamate metabolism, phosphonate and phosphinate metabolism, phosphonate and phosphinate metabolism, glycosaminoglycan biosynthesis—heparan sulfate/heparin.

### 3.3 Establishment of the circRNA‐miRNA and circRNA‐mRNA interaction network

**FIGURE 3:**
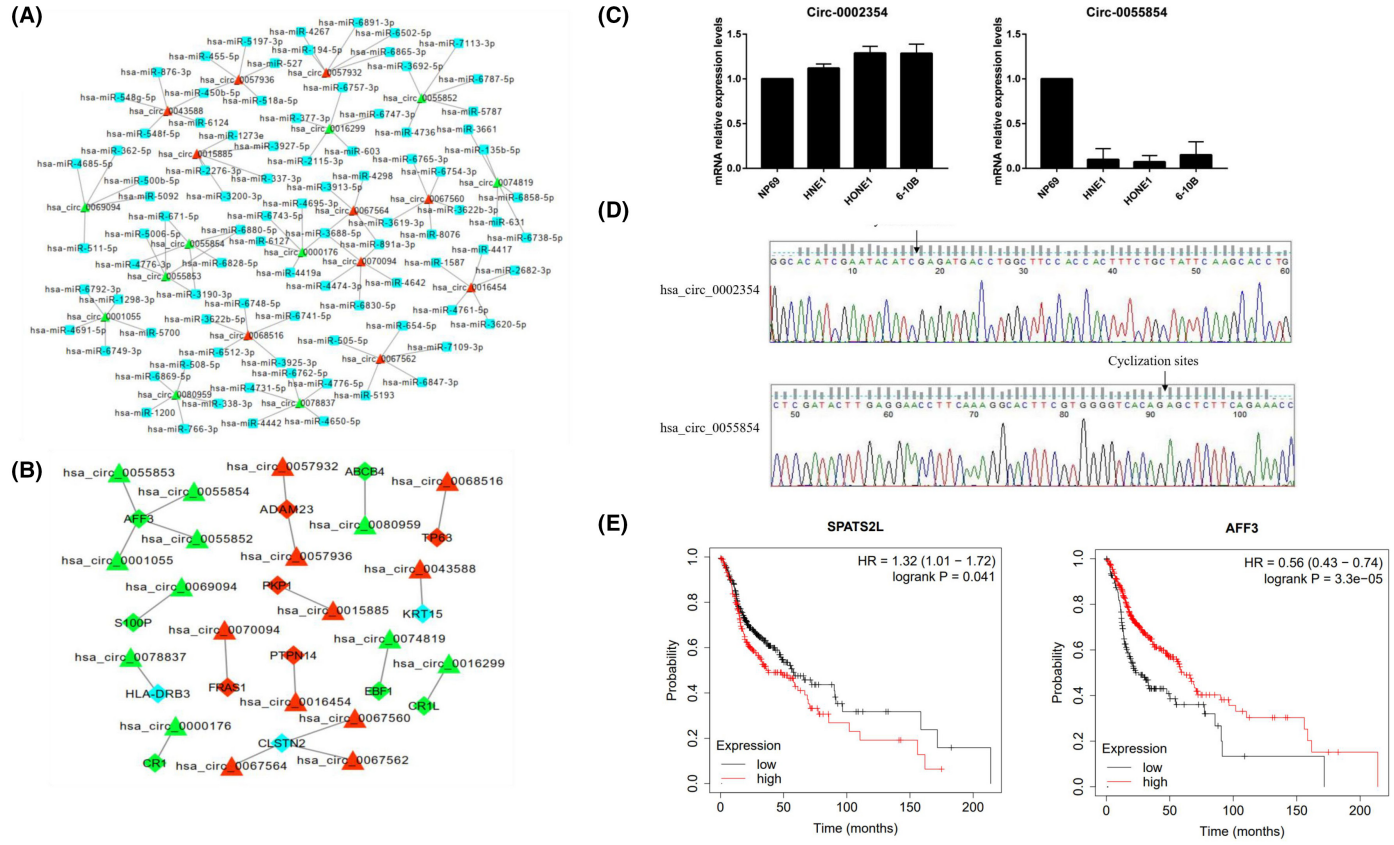
Construction of the circRNA‐miRNA and circRNA‐mRNA co‐expression network. (A) Interactions of the 10 upregulated and downregulated circRNAs with complementary miRNAs targets. (B) Interactions of 10 upregulated and downregulated circRNAs with mRNA. Triangular nodes represent circRNAs, diamond nodes represent mRNAs, and miRNAs are indicated by blue boxes. Red and green represent up‐ and downregulation, and blue represents no different expression. (C) The relative expression of hsa_circ_0002354 and hsa_circ_0055854 in NPC cell lines (HNE1,HONE1,6‐10B) versus control cell line (NP69). Data are mean ± standard deviation. (D) DNA sequencing analysis of the PCR product to confirm the circRNA cyclization site. (E) Prognostic value of SPATS2L and AFF3 in head–neck squamous cell carcinoma (Kaplan–Meier plotter). Low SPATS2L and high AFF3 mRNA expression was significantly correlated with short OS in head–neck squamous cell carcinoma.

To determine the function of circRNAs, the microRNA response elements (MRE) were predicted by using Arraystar data based on TargetScan and miRanda prediction, and five high‐binding potential miRNAs for each circRNA were listed (Supplementary information). We then established a network using Cytoscape to describe interactions of the 10 upregulated and downregulated circRNAs with complementary miRNAs targets (Figure 3A). We got the stress value of every circRNAs by network analysis. The value is calculated by measuring the number of shortest paths passing through a node. hsa_circ_0067564 has the highest stress value. hsa‐miR‐3619‐3p interacted with hsa_circ_0067564 has the highest edge betweenness value. hsa‐miR‐3619‐3p showed significant correlation with small cell carcinoma of the esophagus relapse. Apart from circRNAs with no significant different expression genes, the remaining circRNAs could serve as miRNA sponges indirectly regulating gene expression, namely upregulated circRNAs and upregulated mRNA or downregulated circRNAs and downregulated mRNA respectively (Figure 3B).

Quantitative real‐time reverse transcription polymerase chain reaction (qRT‐PCR) is used to confirm 1 differentially expressed circRNAs expression results randomly selected from microarray analysis. The results showed that hsa_circ_0002354 was significantly upregulated expression in NPC cell lines and hsa_circ_0055854 was significantly downregulated expression in NPC cell lines(Figure 3C) and the amplified PCR product with specific circRNA junctions was directly sequenced using the Sanger sequencing (Figure 3D). SPATS2L is the host gene of hsa_circ_0002354, and AFF3 is the host gene of hsa_circ_0055852. The prognostic value of the mRNA expression of distinct SPATS2L and AFF3 in Head–neck squamous cell carcinoma patients was analyzed by using Kaplan–Meier plotter (http://kmplot.com/analysis/). High SPATS2L (HR = 1.32, *p* = 0.041) and CBX6 (HR = 0.43, *p* = 0.000033) mRNA expression was significantly correlated with short OS in head–neck squamous cell carcinoma patients.

### 3.4 Prediction of *cis*‐target and *trans*‐target genes of the DE lncRNAs

**FIGURE 4:**
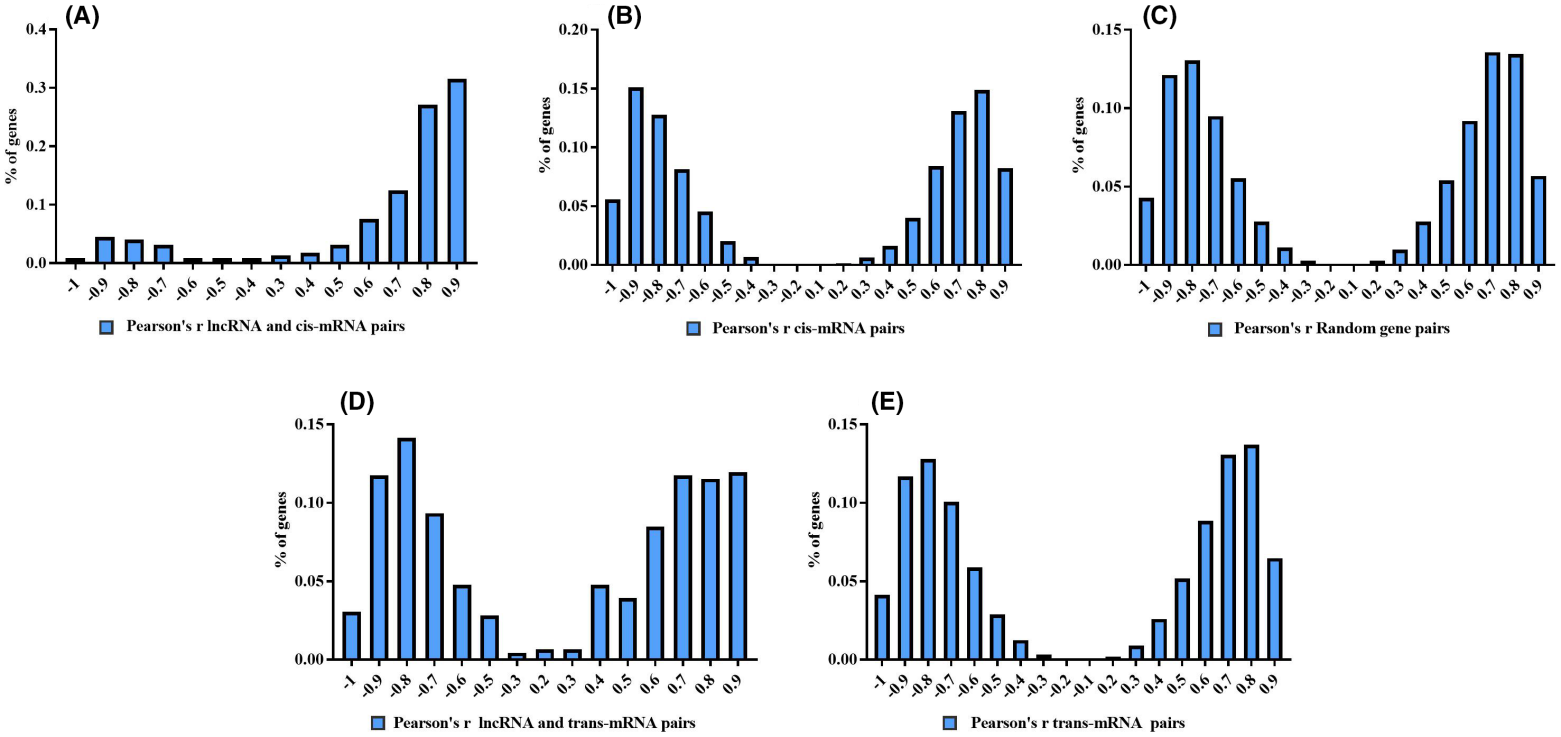
DE lncRNAs cis and trans‐regulatory function. (A) The gene number ratio of PCCs between the expression levels of lncRNA and *cis*‐mRNA. (B) The gene number ratio of PCCs between mRNA pairs. (C) The gene number ratio of PCCs between random mRNA pairs. (D) The gene number ratio of PCCs between the expression levels of lncRNA and *trans*‐mRNA. (E) The gene number ratio of PCCs between the expression levels trans‐mRNA. The values on the *x*‐coordinate is within an interval [*x*, *x* + 0.1] by a constant value.

LncRNAs work in *cis* when its functions close to the chromosome from which they are transcribed, and work in *trans* when they regulate the expression of genes on a different chromosome. To explore DE lncRNAs and DE mRNA potential interactions, we searched for *cis*‐target genes that are physically close to lncRNAs (within 10 kb upstream or downstream). We calculated the Pearson correlation coefficients (PCCs) of three sets: lncRNAs and target genes(*cis*‐mRNAs), *cis*‐mRNAs pairs, and random mRNA pairs. As shown in Figure 4A, lncRNAs and *cis*‐mRNAs pairs showed the strongest correlation (83.55% with absolute Pearson *r* > 0.7; median, 0.86, Figure 4A) than the other two pairs, and are consistent with previous reports suggesting that lncRNAs may be attributed to positive/negative regulatory potentials on neighboring mRNAs.^23^ Interestingly, lncRNAs and *cis*‐mRNAs pairs demonstrated more frequent positive than negative correlations (80.44% vs. 15.11%, respectively; Figure 4A). *cis*‐mRNAs pairs showed stronger correlation (69.64% with absolute Pearson *r* > 0.7; median, 0.78, Figure 4B) than random mRNA pairs (62.17% with absolute Pearson *r* > 0.7; median, 0.75, Figure 4C). In this study, 87 DE lncRNAs have 3904 potential *trans*‐target mRNAs. We computed the PCCS of DE lncRNAs and DE mRNAs to explore the *trans*‐regulatory potential. As shown in Figure 4D, 53.7% DE lncRNAs and DE mRNAs gene pairs showed positive correlation (64.13% with absolute Pearson *r* > 0.7; median, 0.75, Figure 4D). Similarly, 50.9% *trans*‐target mRNAs pairs showed positive correlation (61.83% with absolute Pearson *r* > 0.7; median, 0.77, Figure 4E). These results suggest that the extent of lncRNAs *trans*‐regulatory are similar to DE mRNAs gene pairs.

### 3.5 Construction of lncRNA‐mRNA co‐expression networks

**FIGURE 5:**
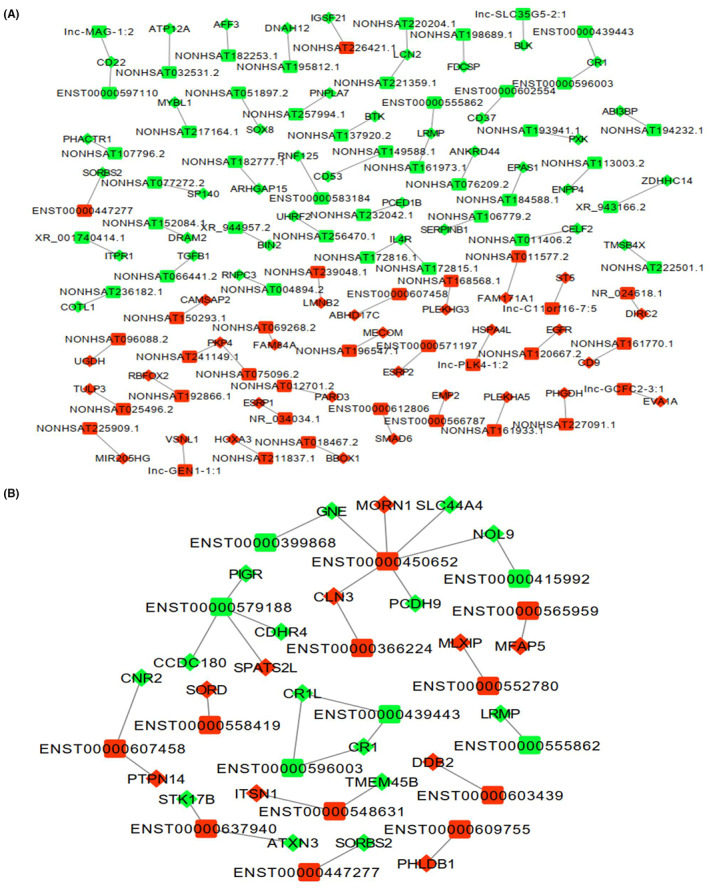
LncRNA‐mRNA co‐expression network. (A) *cis*‐regulatory network. (B) *Trans*‐regulatory network. Diamond and square nodes represent mRNAs and lncRNAs, respectively. Red and green represent up‐ and downregulation, respectively.

To further reveal the potential roles of lncRNAs in NPC progression, lncRNAs and mRNAs that had PCCs ≥0.90 were selected to construct *cis* or *trans* genes network. The *cis*‐regulatory network contains 73 lncRNAs and 67 mRNAs. Seventeen lncRNAs were interacting with 25 mRNAs in the *trans*‐regulatory network (Figure 5A). Thus, it could be seen that many lncRNAs function work through *cis* regulation. Among the 73 *cis*‐regulatory lncRNAs, 71 lncRNAs showed the same expression trend with nearby mRNA, and only two lncRNAs showed the opposite expression trend with neighboring mRNA. Furthermore, most of *cis*‐regulatory lncRNAs have one strongest correlation mRNA. The *trans*‐regulatory network showed that one lncRNA could target many mRNAs and that one mRNA was predicted to be a target of multiple lncRNAs (Figure 5B). The network was complex with some obvious *trans‐*regulation relationships. Vance et al. reported that *trans*‐acting lncRNA could also operate to control nearby gene expression.^24^ Our data showed that *CR1*, *LRMP*, and *SORBS2* are predicted to be mediated not only by *cis*‐acting lncRNA modes of action, but also by *trans*‐acting lncRNA mechanisms.

### 3.6 LncRNAs and circRNAs transcribed from the same gene

LncRNAs and circRNAs are two kinds of endogenous noncoding RNAs. Some studies proved that circRNAs and lncRNAs could be transcribed from the same gene.^25^ Based on the circRNA‐mRNA and lncRNA‐mRNA co‐expression network, we also found that four genes (*CR1*, *AFF3*, *CR1L*, and *PTPN14*) could be transcribed into circRNAs and lncRNAs (Table 3). lncRNAs and circRNAs showed the same expression trend in lncRNA‐mRNA‐circRNA network from the same parental gene.

**TABLE 3:** LncRNAs and circRNAs transcribed from the same gene.

Gene	Location	lncRNA	circRNA
CR1	1q32.2	ENST00000439443, ENST00000596003	hsa_circ_0000176
AFF3	2q11.2	NONHSAT182253.1	hsa_circ_0055852, hsa_circ_0001055, hsa_circ_0055853, hsa_circ_0055854
CR1L	1q32.2	ENST00000439443, ENST00000596003	hsa_circ_0016299
PTPN14	1q41	ENST00000607458	hsa_circ_0016454

### 3.7 The diagnostic value of key mRNAs

**FIGURE 6:**
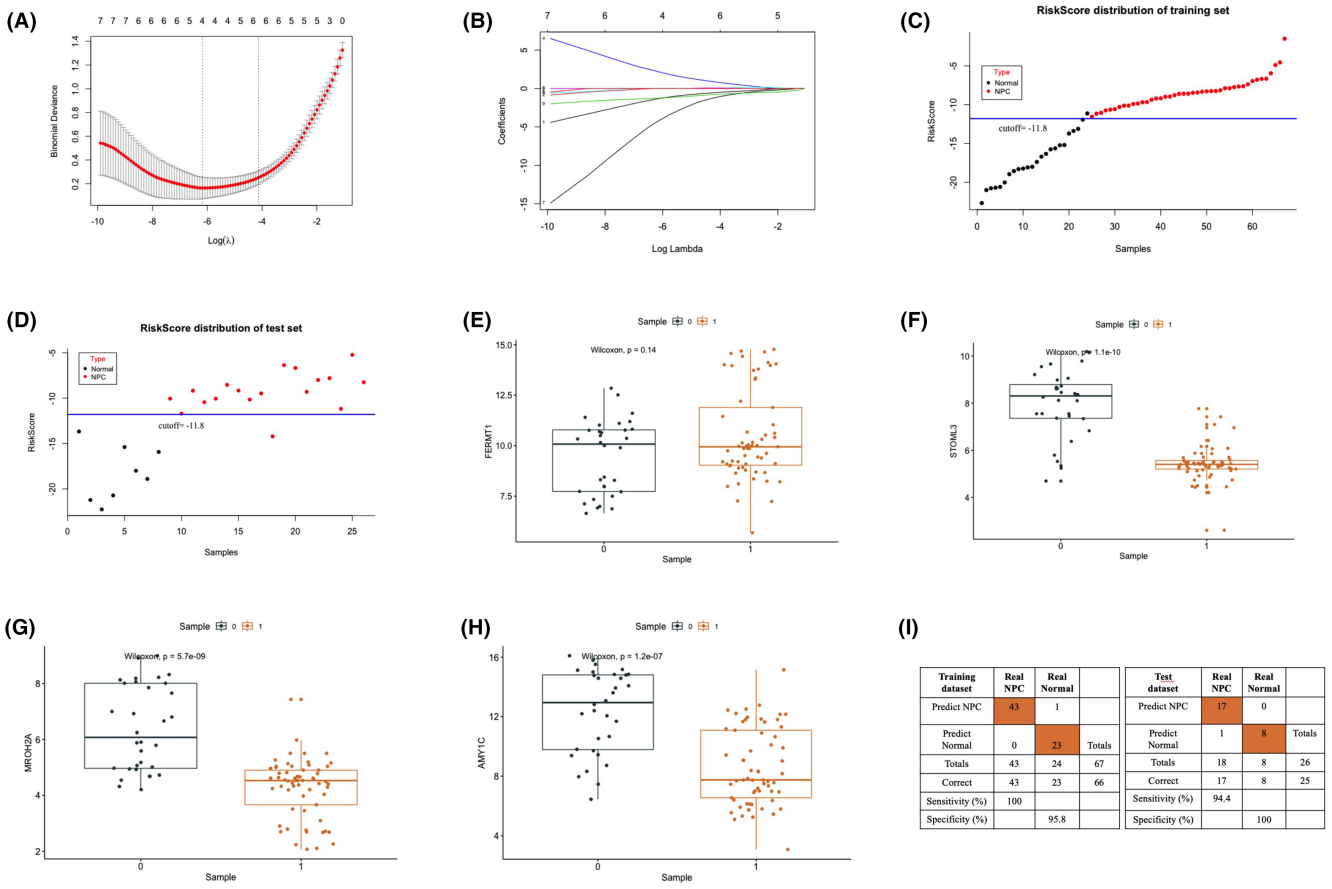
Diagnostic model and gene expression between NPC and normal tissues. (A) Binomial deviance was plotted versus log(Lambda). The vertical dotted line indicates the lambda value with the minimum error and the largest lambda value. (B) LASSO coefficient profiles of the genes at varying log‐transformed lambda values. (C) The risk score distribution on the basis of the four genes signature of training set. (D) The risk score distribution on the basis of the four genes signature of test set. The significance tests were carried out using *Wilcoxon* test and *p‐value* of each gene was presented. (E) *FERMT1*, (F) *STOML3*, (G) *MROH2A*, (H) *and AMY1C*. (I) Confusion tables of binary results of the diagnostic prediction model in the training and test data sets.

We constructed lasso regression analysis using nine genes by R language. A four‐gene diagnostic risk model was built: 0.7889 *FERMT1 −1.4967 *STOML3 −1.0247 *MROH2A −0.5226 *AMY1C. As shown in Figure 6A–D, the model discrimination information started to stabilize after the inclusion of four to six genes. *STOML3*, *MROH2A*, and *AMY1C* were differentially expressed in NPC samples as compared to normal samples with statistical significance (*p < 0.01*; Figure 6F–H) except for *FERMT1* (Figure 6E).The sensitivity and specificity of RiskScore were calculated using the score threshold at −11.8 to identify NPC and we found that the RiskScore has 94.4% sensitivity and 100% specificity as compared with clinical diagnosis in test data set (Figure 6I).

## 4 DISCUSSION

Nasopharyngeal carcinoma is one of the most common malignant disease in southern China. The molecular mechanisms underlying the regulation remain incompletely understood. This study, to the best of our knowledge, presents the first simultaneous investigation of circRNAs, mRNAs, and lncRNAs in clinical NPC tissues using a microarray analysis. A total of 3048 circRNAs, 2179 lncRNAs, and 2015 mRNAs were observed to be dysregulated.

Emerging evidence revealed that lncRNAs may serve as a required novel biomarker and therapeutic target. To explore the expression profiles and functions of lncRNAs, we performed a genome‐wide analysis of lncRNAs. The number of upregulated DE lncRNAs was more than twice than those downregulated. LncRNAs have been proposed to carry out transcriptional regulation either in *cis*‐acting (on neighboring genes) or in *trans*‐acting (on distantly located genes).^26^, ^27^ The *cis*‐target gene GO term results were closely related to NPC clinical characterizations, such as glossopharyngeal nerve development. It was also reported that hypoglossal nerve palsy on presentation was caused by locally advanced NPC.^28^ The top MF terms, calcium‐release channel activity related genes, might be associated with the development of NPC.^29^ The *trans*‐target genes were associated with L‐methionine and L‐serine. Study have shown that deregulation of methionine metabolism was a determinant of progression and prognosis of hepatocellular carcinoma.^30^ Our results implied an important functional role of L‐methionine and L‐serine in NPC.

We applied bioinformatic analysis to explore the potential correlations between lncRNA and mRNA. 80.44% of the pairs of lncRNAs and *cis*‐mRNAs pairs demonstrated positive correlations. The result means that lncRNAs and *cis*‐mRNAs have the same direction effects and conform to the ceRNA function mechanism. Some studies showed that the expression of both protein‐coding genes and lncRNA genes tended to be more positively correlated than negatively correlated in *trans*‐regulatory relationships.^31^, ^32^ Here, we found that 53.7% of the pairs of lncRNAs and *trans*‐mRNAs pairs showed positive correlations. To gain insight into how interactions between lncRNAs and their target genes, PCCs ≥0.90 between them were selected to construct *cis* or *trans* genes network. The *cis*‐regulatory network contained 73 lncRNAs and the *trans*‐regulatory network contained 17 lncRNAs. This suggested that lncRNA‐mediated *cis* regulation is a prevalent regulatory mode.^33^ In general, *cis*‐regulatory lncRNAs regulate the transcription of nearby genes in *cis* by recruiting remodeling factors to local chromatin or facilitating access to enhance and promote for transcriptional machinery molecules.^27^, ^34^ However, two lncRNAs showed the opposite expression trend with neighboring mRNA in *cis*‐regulatory network. Further studies on the opposite expression trend are needed to verify the relationship.

Recent some studies have shown that circRNAs can sponge miRNAs have been reported in NPC. For instances, Hong et al.^35^ reported that circCRIM1 interacts with miRNAs by acting as ceRNA to promote NPC metastasis and chemoresistance through upregulating *FOXQ1*. Li et al.^36^ reported that circCTDP1/miR‐320b promote NPC progression via regulating the expression of homeobox A10. Wei et al.^37^ reported that circ_0008450 acts as an oncogene in NPC cells through regulating miR‐577/*CXCL9* signaling. In this study, we found that the expression of circRNAs and RNA transcripts have the same trend. These data are in line with ceRNA hypothesis.

Lin et al. selected four cell lines and four patients with nasopharyngeal carcinoma (NPC; two of which were metastatic NPC). They constructed ceRNA and performed differential expression analysis, and they found CDKN1B, ZNF302, ZNF268, and other key differential genes.^14^ In this study, we used microarrays to detect differential expression (DE) of circRNAs, mRNAs, and lncRNAs in nasopharyngeal carcinoma (NPC) tissues. We also performed network analyses (competitive endogenous RNA network analysis for circRNAs, mRNAs, and miRNAs, and co‐expression network analysis for lncRNAs and mRNAs) trying to infer the potential interactions of these functional RNAs. Our selected differential genes were compared with those of Lin et al. in cancer and noncancer cell lines, and some of the results were found to be identical, such as MBNL2, as well as some of their previously unreported differential expressed genes, which may complement the database of noncoding RNA. In addition, we built a diagnostic model of NPC using DE mRNAs identified in this study.

The circRNAs number of *DOCK1* gene is highest than other genes. *DOCK1* is one of the dedicators of cytokinesis (DOCK) family proteins that are evolutionary conserved exchange factors for the Rho GTPases Rac and Cdc42.^38^ Previous studies have shown that these proteins have widely involved in cancer, disease of central nervous, and immune systems.^38^, ^39^ A growing number of studies have shown that the dysregulated *DOCK1* gene plays diverse roles in cellular processes. DOCK1–Rac signaling as an HER2 effector pathway is essential for HER2‐mediated breast cancer progression to metastasis.^39^ Subsequent studies found that downregulation of *DOCK1* could increase the chemosensitivity in bladder cancer cells through preventing epithelial–mesenchymal transition.^40^ *DOCK1* overexpression is closely associated with poor prognosis in acute myeloid leukemia.^41^ Taking the literatures and our data together, *DOCK1* might be an important prognostic marker in NPC, despite the underlying functions need further investigation.

We found that four genes (*CR1*, *AFF3*, *CR1L*, and *PTPN14*) could be transcribed into circRNAs and lncRNAs. *CR1* gene is a member of the receptors of complement activation family and plays an important role in many different cell types, particularly in the circulatory system.^42^ Some studies reported that *CR1* gene is a secondary receptor for Epstein–Barrvirus (EBV) and can expedite the entry of EBV into cells.^43^, ^44^ The clinical use of EBV as an etiological biomarker in NPC is well documented, especially in undifferentiated NPC. Therefore, lncRNA and circRNA from *CR1* are likely to be contributing factors to the pathogenesis of NPC introduced by EBV. *AFF3* gene was first considered a lymphoid‐specific gene and located in the nuclear of B cells. *AFF3* upregulation mediates tamoxifen resistance and estrogen‐independent growth in breast cancer.^45^ *AFF3* is a mediator of the oncogenic effects of β‐catenin involved in adrenocortical carcinoma, acting on transcription and RNA splicing.^46^ However, the underlying mechanism of *AFF3* in NPC remains unclear, and we are currently investigating it.

We identified four novel gene signatures associated with the diagnosis of NPC patients based on the microarray analysis, containing *STOML3*, *MROH2A*, *AMY1C*, and *FERMT1*. *FERMT1*, and *STOML3* have been reported to be related to epithelial–mesenchymal transition.^47^, ^48^ Four genes along with the regression coefficients were selected and a formula was constructed to act as a risk score model for the diagnosis of NPC.

This study has several limitations. First, the sample size analyzed here was relatively small and represented a part of the noncoding RNA (ncRNA) expression pattern. Larger numbers of clinical samples combined with improved analysis methods will be of considerable interest. Second, the potential functions of these ncRNA were explored using bioinformatics analysis. Great efforts should be given to research the functions and mechanisms of these ncRNA with in vitro and in vivo experiments. Third, we hope that the RiskScore model should be further studied in additional clinical samples and serve as new biomarkers for the diagnosis of NPC.

## 5 CONCLUSIONS

The present study presented a pattern of dysregulated circRNAs, mRNAs, and lncRNAs in NPC, as determined by microarray analysis. The results suggest that regulatory interactions with cis‐acting, rather than with trans‐acting, could be the major mechanisms of action for NPC. What is more, the expression of circRNAs and RNA transcripts have the same trend. DOCK1, CR1, and AFF3 genes may play an important functional role in the pathogenesis of NPC. In addition, we screened out the first 10 upregulated and downregulated circRNAs, lncRNAs, and mRNAs, such as hsa‐circ‐0067562, NONHSAT232922.1, and HOXB13 (Tables S1–S3). The RiskScore model can identify NPC cases with a high sensitivity and specificity. Our study provides a theoretical basis for using circRNAs, mRNAs, and lncRNAs to generate meaningful hypotheses for future studies.

## AUTHOR CONTRIBUTIONS

Feifan Guo and Dayang Chen contributed equally to this work in the design and write original draft should be considered as co‐first authors. Zengyan Zong and Wei Wu collected and analyzed the data. Chan Mo and Zhou Zheng performed the literature search and were responsible for data visualization. Jian Li collected clinical samples and related medical records. Xiuming Zhang and Dan Xiong designed and revised the study carefully and rigorously. All authors reviewed the results and approved the final version of the study.

## FUNDING INFORMATION

This study was supported by the National Natural Science Foundation of China (Grant NO. 81772921). A grant from the Science and Technology Planning Project of Shenzhen City of China (JCYJ20190812171816857). Shenzhen Key Medical Discipline (NO. SZXK054).

## CONFLICT OF INTEREST

The authors declare no conflict of interest.

## Supporting information


Table S1

Figure S1

Figure S2

Figure S3

## Data Availability

The data that support the findings of this study are available from the corresponding author upon reasonable request.
