# Supplementary material for: Comprehensive analysis of aberrantly expressed circRNAs, mRNAs and lncRNAs in patients with nasopharyngeal carcinoma
Source: J Clin Lab Anal. 2023 Jan 4;37(2):e24836. doi: 10.1002/jcla.24836 (PMC9937882; doi:10.1002/jcla.24836)
Supplement: Supplementary file 1 — Table S1 [file JCLA-37-e24836-s004.docx]

**Supplementary Table 1. Significant differentially expressed circRNAs in NPC tissues compared with the chronic nasopharyngitis tissues.**

| The Upregulated (Fold Change) | The Downregulated (Fold Change) |
| --- | --- |
| hsa_circ_0067562 | hsa_circ_0078837 |
| hsa_circ_0070094 | hsa_circ_0000176 |
| hsa_circ_0067564 | hsa_circ_0055852 |
| hsa_circ_0015885 | hsa_circ_0001055 |
| hsa_circ_0067560 | hsa_circ_0069094 |
| hsa_circ_0057936 | hsa_circ_0055853 |
| hsa_circ_0057932 | hsa_circ_0016299 |
| hsa_circ_0016454 | hsa_circ_0080959 |
| hsa_circ_0043588 | hsa_circ_0074819 |
| hsa_circ_0068516 | hsa_circ_0055854 |

**Supplementary Table 2. Significant differentially expressed lncRNAs in NPC tissues compared with the chronic nasopharyngitis tissues.**

| The Upregulated (Fold Change) | The Downregulated (Fold Change) |
| --- | --- |
| NONHSAT232922.1 | lnc-TTC8-4:3 |
| NONHSAT008998.2 | NONHSAT204179.1 |
| lnc-GEN1-1:1 | lnc-OMG-1:2 |
| NR_024615.1 | NR_027068.1 |
| lnc-MAP1LC3B2-14:3 | NR_003063.1 |
| NR_034034.1 | ENST00000597110 |
| NONHSAT225909.1 | NONHSAT245356.1 |
| NONHSAT216172.1 | NONHSAT154013.1 |
| NONHSAT241213.1 | XR_001738662.1 |
| NONHSAT230855.1 | NR_131757.1 |

**Supplementary Table 3. Significant differentially expressed mRNAs in NPC tissues compared with the chronic nasopharyngitis tissues.**

| The Upregulated (Fold Change) | The Downregulated (Fold Change) |
| --- | --- |
| HOXB13 | LTF |
| DHRS2 | C20orf85 |
| LCE1C | C1orf194 |
| ERC2 | MORN5 |
| TMEM211 | AKAP14 |
| HOXC6 | C11orf97 |
| HOXC8 | GSTA3 |
| HOXA10 | C9orf171 |
| CLSTN2 | CDHR4 |
| BBOX1 | LDLRAD1 |
