# Supplementary figures and images for: Comprehensive analysis of aberrantly expressed circRNAs, mRNAs and lncRNAs in patients with nasopharyngeal carcinoma
Source: J Clin Lab Anal. 2023 Jan 4;37(2):e24836. doi: 10.1002/jcla.24836 (PMC9937882; doi:10.1002/jcla.24836)

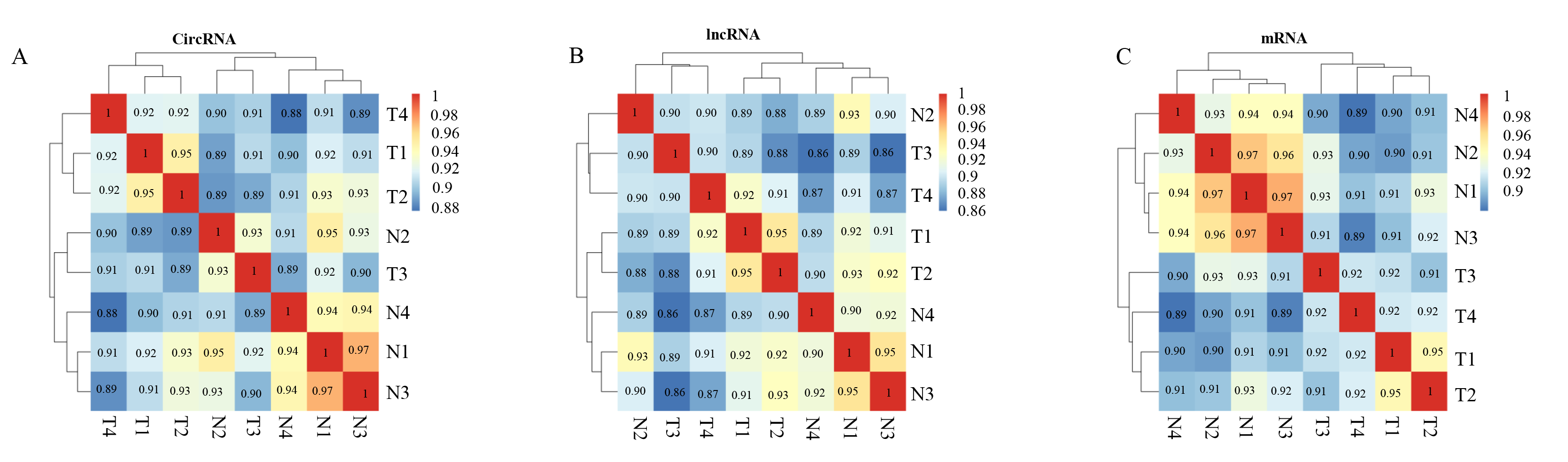

Supplement: Supplementary file 2 — Figure S1 [file JCLA-37-e24836-s003.tif]

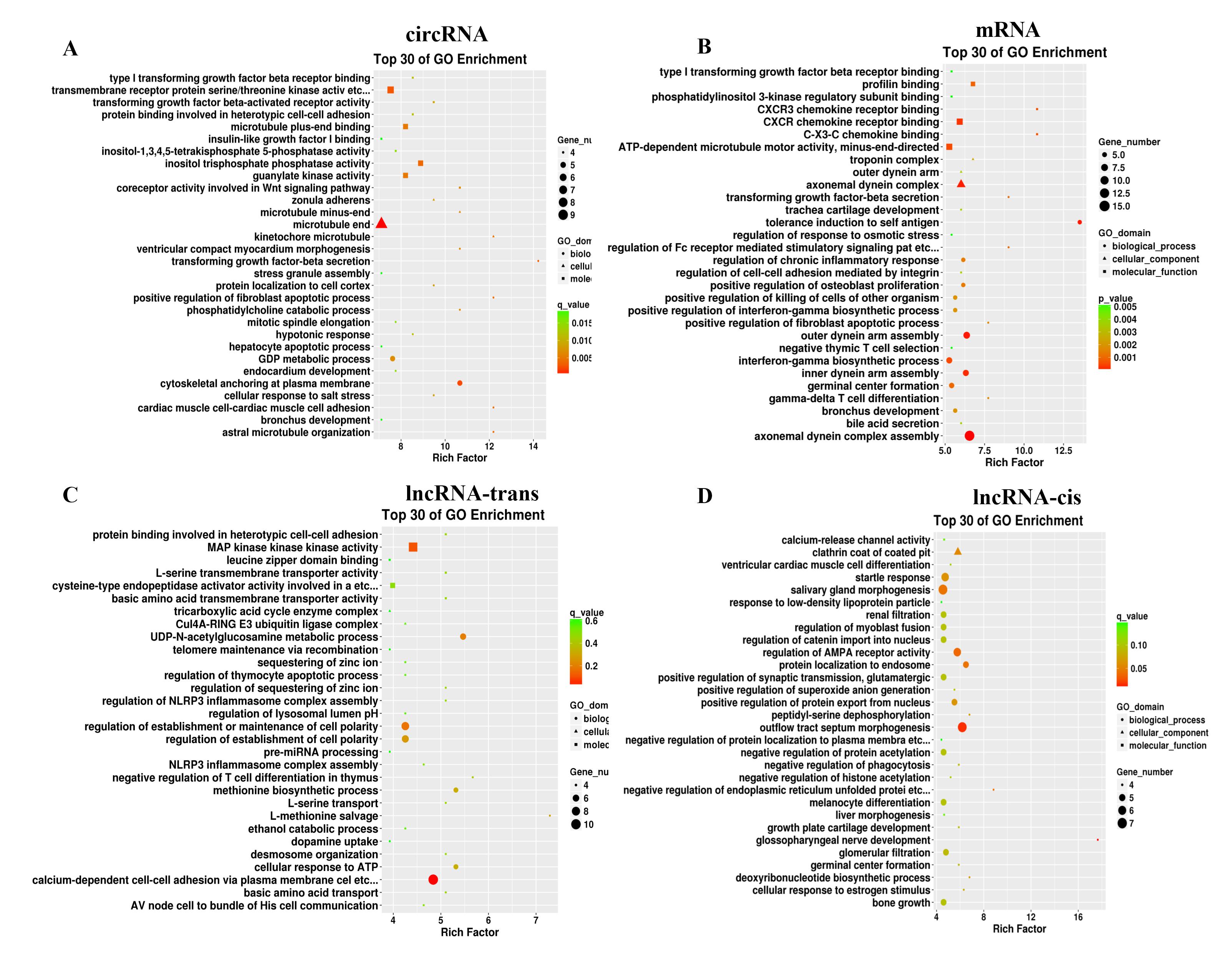

Supplement: Supplementary file 3 — Figure S2 [file JCLA-37-e24836-s001.jpeg]

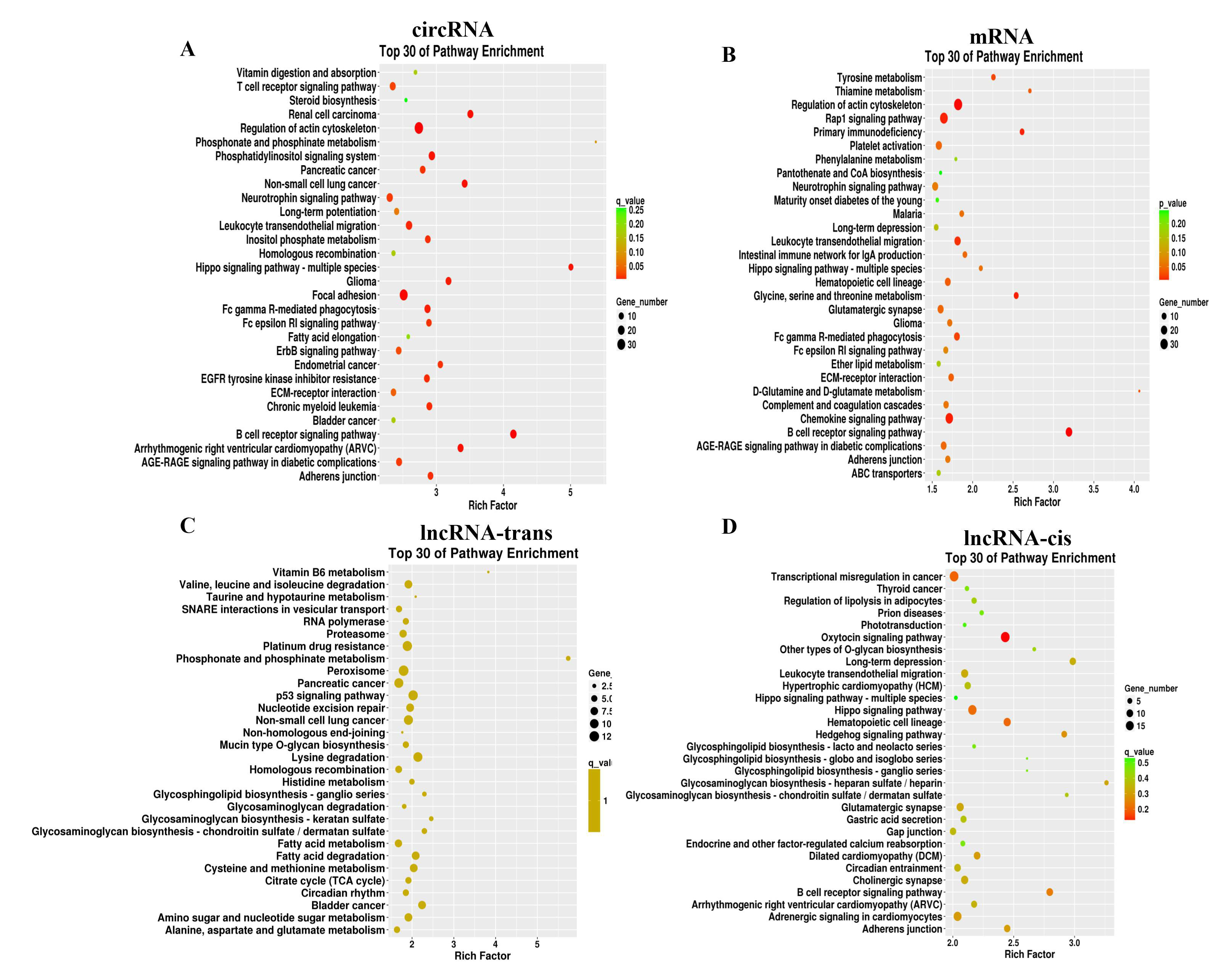

Supplement: Supplementary file 4 — Figure S3 [file JCLA-37-e24836-s002.tif]
